# Comorbidities in heart failure patients that predict cardiovascular readmissions within 100 days—An observational study

**DOI:** 10.1371/journal.pone.0296527

**Published:** 2024-01-02

**Authors:** Mia Scholten, Jason Davidge, Björn Agvall, Anders Halling

**Affiliations:** 1 Center for Primary Health Care Research, Department of Clinical Sciences, Lund University, Lund, Sweden; 2 Capio Vårdcentral Halmstad, Halmstad, Sweden; 3 Department of Research and Development, Region Halland, Halmstad, Sweden; BSMMU: Bangabandhu Sheikh Mujib Medical University, BANGLADESH

## Abstract

**Background:**

Heart failure (HF) commonly arises as a complication to cardiovascular diseases and is closely associated with various comorbidities. The impacts of these comorbidities in patients with HF are diverse. We aimed to analyze the increased risk for cardiovascular-related readmission within 100 days after discharge in patients with HF depending on their different comorbidities.

**Methods:**

A population-based retrospective study was conducted in Region Halland with 5029 patients admitted to hospital with a diagnosis of HF during 2017–2019. The occurrence and number of comorbidities were recorded. Competing risk regression was employed to analyze the hazard ratio (HR) of 10 comorbidities for cardiovascular-related readmission within 100 days after discharge. A composite measure of the 10 common comorbidities was constructed with the comorbidities as dichotomous indicator variables and Rasch analysis. Receiver operating characteristic (ROC) and area under curve (AUC) after logistic regression were used to estimate how well the model explained the probability of death or readmission within 100 days after discharge according to their individual comorbidity level.

**Results:**

HF patients with atrial fibrillation, chronic obstructive pulmonary disease, chronic kidney disease, peripheral artery disease or diabetes mellitus as comorbidities had an increased HR for readmission within 100 days after discharge. When these comorbidities were adjusted together, only atrial fibrillation, chronic kidney disease and chronic obstructive pulmonary disease had an increased HR for readmission. ROC analysis after the most complete models using logistic regression with the comorbidities as dichotomous indicator variables or Rasch analysis had a low AUC.

**Conclusions:**

Atrial fibrillation, chronic kidney disease or chronic obstructive pulmonary disease were significantly associated with increased risk for readmission in HF patients, but ROC analysis showed a low AUC, which indicates that other factors are more important for predicting the increased risk of readmission.

## Introduction

HF is associated with multimorbidity [1], but different subgroups of HF are reported to have varying comorbidities [2]. These comorbidities could exacerbate HF and consequently increase the need for hospitalizations. Most of the comorbidities share risk factors resulting in various degrees of impairment and need for hospitalization in HF patients. However, some of the common comorbidities are attributable to HF, which appear early or after long-term exposure. Common comorbidities in HF patients such as atrial fibrillation, peripheral artery disease (PAD), cerebrovascular insults (CVI), valvular heart disease, ischemic heart disease, acute myocardial infarction, chronic kidney disease (CKD), diabetes mellitus, chronic obstructive pulmonary disease (COPD) and hypertension are usually linked with each other in the pathophysiology contributing to HF diagnosis.

HF is a prevalent comorbidity in patients with diabetes mellitus, as indicated by scientific studies [3, 4]. The heightened prevalence of HF in diabetes mellitus patients remains significant even after adjusting for coronary heart disease and its associated risk factors, including age, gender, race, smoking, physical inactivity, obesity, hypertension, and hyperlipidemia [3]. The diabetic myocardium has a typical characteristic of left ventricular concentric remodeling, promoting impaired myocardial metabolism and systolic dysfunction [4, 5]. The subsequent increase in myocardial stiffness may translate to diastolic dysfunction, atrial enlargement and valvular heart disease, which facilitates the incidence of atrial fibrillation in patients with diabetes mellitus [5–8]. Diabetes mellitus has been reported as a predictor of cardiovascular mortality or HF hospitalization, particularly among HF patients with high HbA1c [3, 9]. After adjustment for age, gender, a 1-mmol/L-rise of fasting plasma glucose was associated with a 1.10-fold-increased risk of HF hospitalization [10]. Furthermore, those patients with diabetes mellitus tend to have a poorer prognosis compared to patients without [11].

COPD patients have an elevated risk of developing HF due to shared risk factors like smoking, age, and inflammation [14, 15]. The prevalence of HF precursors, such as diabetes, atrial fibrillation, hypertension, and ischemic heart disease, is higher in COPD patients [16–19]. Those with both HF and COPD have a higher mortality rate compared to those with only one these conditions [15]. A meta-analysis reported that COPD was associated with an increased risk of all-cause hospitalization and HF specific hospitalization in the chronic HF population [12].

In a Canadian study of elderly HF patients, 5.6% were readmitted within seven days and 18% were readmitted within 30 days after hospital discharge. The readmission rates increased significantly with advancing age and were associated with comorbidities including kidney disease [13].

A previous European study examined the presence of comorbidites in 3226 outpatients diagnosed with chronic HF. The comorbidities considered included CKD, anemia, CVI, hyper- and hypothyroidism, COPD, sleep apnea and diabetes mellitus. These comorbidities were independently associated with higher age, NYHA functional class, heart rate, ischemic etiology of HF, hypertension, and atrial fibrillation [14]. The most prevalent comorbidities observed in the study group were CKD, anemia and DM, which were all strongly associated with higher mortality rates and/or HF hospitalization [14].

It has been previously reported that the increased risk for cardiovascular-related readmission within 100 days after discharge in HF patients in southern Sweden was associated with advanced age, hospital stay > 6 days, renal impairment, elevated heart rate and higher N-terminal-pro Brain Natriuretic Peptide (NT-proBNP) levels [23]. The likelihood of readmission decreased when a combination of beta-blockers and renal-angiotensin-aldosterone-system inhibitors was administered alongside an echocardiography performed upon admission.

The present study aims to determine the extent to which 10 common comorbidities affect the risk for cardiovascular-related readmission within 100 days after discharge in HF patients.

## Methods

### Setting and study population

Region Halland is located in south-western Sweden and has an estimated population of 320,000 inhabitants. There are three acute care hospitals, 40 inpatient wards, two emergency departments, 30 specialized outpatient clinics and 48 healthcare providers in primary care. This is a retrospective population-based study in Region Halland, encompassing patients who were hospitalized with a diagnosis of HF between 2017 and 2019. The data extraction took place between the 1^st^ of September 2020 and the 1^st^ of June 2021 for research purposes. A total of 5029 individuals were admitted for HF and subsequently discharged. The clinical characteristics were recorded from the date of admission until 100 days following their post-discharge.

### Data source and measurements

The data for this study were sourced from the Regional Healthcare Information Platform (RHIP) administered by Region Halland. RHIP comprises comprehensive data from both primary healthcare, including private and public healthcare providers, and secondary healthcare levels. The database incorporates comprehensive healthcare information, spanning healthcare utilization, health economics, staff utilization, pharmacotherapy and various chronic diseases including HF. Data comprising ICD-diagnoses, laboratory samples and examinations undergone by each patient within Region Halland are accessible, but no information about the severity of each condition or results of treatments. A detailed description of the database can be found in a previous publication [15]. Within this HF cohort, every echocardiography has been successfully conducted and the ejection fraction has been established in 99% of the patients, enabling determination of the HF-subgroup in these individuals [24]. The authors did not have access to data that could identify individual participants during or after data collection. The data used in this study were pseudo-anonymized, which means that the participants’ identities were concealed from the researchers. However, through specific administrative processes, the identities could potentially be revealed. In this study, no participant identities were disclosed to the researchers.

The study participants were enrolled if they were hospitalized with an ICD-10 diagnosis of HF according to Table 1 in S1 Appendix, and subsequently discharged with a HF diagnosis. The registered comorbidities, which were collected during the lookback period from the 1^st^ of January 2013 until the 31^st^ of December 2019, included: hypertension, ischemic heart disease, acute myocardial infarction, CVI, atrial fibrillation, diabetes mellitus, valvular heart disease, COPD, PAD, CKD, until they were hospitalized (Table 1). A patient could only be included once in the study. For those patients admitted to hospital more than once during the study period, only the first hospitalization was included. Readmission due to a cardiovascular disease within 100 days after discharge was registered. Within the study period of 2017–2019 at RH, a total of 7436 patients were identified with HF. Among them, 5494 had a hospital admission for HF, and 465 patients died before discharge. Consequently, the study included 5029 patients who were hospitalized for HF and subsequently discharged with a confirmed HF diagnosis. A flowchart for the study procedure is displayed in Figure (S1 Appendix).

**Table 1:** Prevalence of heart failure patients and their comorbidities within different age groups, subgroups and levels of renal function.

	HF	AF	PAD	CVI	VHD	HTN	IHD	AMI	CKD	DM	COPD
	n (%)	n (%)	n (%)	n (%)	n (%)	n (%)	n (%)	n (%)	n (%)	n (%)	n (%)
***Age (years)***											
<50	99 (1)	18 (1)	1(0)	1 (0)	9 (1)	22 (1)	19 (1)	16 (2)	9 (1)	16 (1)	1 (0)
50–75	1416 (28)	645 (22)	63 (25)	160 (20)	248 (24)	923 (24)	662 (29)	357 (37)	257 (22)	460 (35)	303 (33)
>75	3514 (70)	2243 (77)	184 (74)	641 (80)	787 (75)	2831 (75)	1633 (71)	587 (61)	900 (77)	855 (64)	604 (67)
P-value		<0.001	<0.001	<0.001	<0.001	<0.001	<0.001	<0.001	<0.001	<0.001	<0.001
***HF—subgroup***											
HFpEF	1147 (23)	704 (24)	56 (23)	164 (20)	369 (35)	924 (24)	474 (20)	199 (21)	290 (25)	301 (23)	238 (26)
HFmrEF	898 (18)	487 (17)	45 (18)	140 (17)	193 (18)	637 (17)	525 (23)	286 (30)	205 (18)	243 (18)	142 (16)
HFrEF	1010 (20)	540 (19)	58 (23)	132 (16)	215 (21)	662 (18)	581 (25)	273 (28)	239 (21)	291 (22)	133 (15)
HF-NDP	1974 (40)	1175 (40)	89 (36)	366 (46)	267 (26)	1553 (41)	734 (32)	202 (21)	432 (37)	496 (37)	395 (44)
P-value		<0.001	<0.001	<0.001	<0.001	<0.001	<0.001	<0.001	<0.001	<0.001	<0.001
***Renal function (ml/min)***											
eGFR ≥ 60	1826 (36)	880 (30)	69 (28)	268 (33)	339 (32)	1184 (31)	783 (34)	377 (39)	68 (6)	445 (33)	375 (41)
eGFR 30–59	2437 (49)	1542 (53)	128 (52)	408 (51)	551 (53)	1934 (51)	1160 (50)	438 (46)	542 (46)	622 (47)	422 (46)
eGFR <30	753 (15)	481 (17)	50 (20)	124 (15)	154 (15)	652 (17)	364 (16)	142 (15)	556 (48)	263 (20)	110 (12)
P-value		<0.001	<0.001	<0.001	<0.001	<0.001	<0.001	<0.001	<0.001	<0.001	<0.001

Note; HF = heart failure, HFrEF = heart failure with reduced ejection fraction, HFmrEF = heart failure with mildly reduced ejection fraction, HFpEF = heart failure with preserved ejection fraction, HF—NDP = heart failure with no defined subgroup, AF = atrial fibrillation, CKD = chronic kidney disease, VHD = valvular heart disease, PAD = peripheral artery disease, IHD = ischemic heart disease, AMI = acute myocardial infarction, CVI = cerebrovascular insult, VHD = valvular heart disease, COPD = chronic obstructive pulmonary disease, DM = diabetes mellitus, HTN = hypertension, eGFR = estimated glomerular filtration rate (ml/min).

The HF-subgroups were divided into HF with preserved ejection fraction (HFpEF), HF with mildly reduced ejection fraction (HFmrEF), HF with reduced ejection fraction (HFrEF), and HF with no defined subgroup (HF-NDP) [16]. NT-proBNP was used as a biomarker for HF. The recorded NT-proBNP values were retrieved from the time of hospitalization and the highest values during the period seven days before the index and throughout the hospitalization. NT-proBNP levels were measured at a new onset or acute worsening of HF symptoms and further divided into three groups to determine the probability of HF in different age groups: A NT-proBNP value < 300 ng/L was considered normal and defined as HF unlikely; Elevated values were defined depending on the patient age as grey-zone or HF likely (Table 2 in S1 Appendix) [17]. Renal function was determined by eGFR (ml/min/1.73 m^2^). Renal function was defined as normal when eGFR ≥ 60 ml/min, lowered when eGFR was 30–59 ml/min or impaired when eGFR < 30 ml/min.

### Statistical analyses

The prevalence of 10 common comorbidities were compared among HF patients based on age, HF-subgroup and levels of renal function. Frequencies, percentages and cross-tabulations were used for descriptive analysis and Chi-square-test was used to calculate the p-values. P-value ≤ 0.05 was considered statistically significant.

Primary outcome was readmission due to cardiovascular-related events within 100 days after discharge. The 100-day follow-up after hospital discharge with HF was chosen since it is considered as the most vulnerable period. Competing risk regression was used to estimate the HR of all HF patients with and without 10 common comorbidities for cardiovascular-related readmission within 100 days after discharge, which even considered the mortality during the study period. The HR was stratified for age, gender, HF-subgroup, levels of NT-proBNP and renal function. The comorbidities in HF patients with statistically significant HR were further adjusted in the same model to compare with the HRs for these comorbidities separately.

ROC and AUC were used to estimate how well the model explained the probability of death or readmission within 100 days after discharge according to their individual comorbidity level calculated by logistic regression or Rasch analysis [18]. Linear predictions were made based on models by adding variables in steps (models xb1—xb6), e.g. comorbidities, age, gender, HF-subgroup, NT-proBNP, renal function. Comorbidities were included as a composite measure that had been constructed using logistic regression or Rasch analysis (Table 3 in S1 Appendix).

All calculations and graphs were performed with STATA version 17.0 (Stata Corporation, Texas, USA).

## Ethics

The Swedish Ethical Review Authority, Stockholm Department 2 Medicine, granted approval to conduct the study under registration number 2020–00455. The requirement for informed consent was waived, which received approval from the Swedish Ethical Review Authority, Stockholm Department 2 Medicine. All the methods in this study were carried out in accordance with relevant guidelines and regulations.

## Results

The total prevalence of the comorbidities in HF patients was 58% for atrial fibrillation, 5% for PAD, 16% for CVI, 21% for valvular heart disease, 75% for hypertension, 46% for ischemic heart disease, 19% for acute myocardial infarction, 23% for CKD, 26% for diabetes mellitus and 18% for COPD (Table 1). Almost all the HF patients with atrial fibrillation, ischemic heart disease, PAD, CVI, valvular heart disease, acute myocardial infarction, hypertension, CKD, diabetes mellitus or COPD as comorbidity were over 50 years old (Table 1). Most HF patients with acute myocardial infarction (61%), diabetes mellitus (64%), COPD (67%), ischemic heart disease (71%), PAD (74%), valvular heart disease (75%), hypertension (75%), atrial fibrillation (77%), CKD (77%) or CVI (80%) as comorbidity were over 75 years of age (Table 1).

HF patients with atrial fibrillation (HR 1.22, 95% CI 1.09–1.37), COPD (HR 1.17, 95% CI 1.03–1.34), CKD (HR 1.29, 95% CI 1.12–1.48), PAD (HR 1.28, 95% CI 1.03–1.61) or diabetes mellitus (HR 1.13, 95% CI 1.00–1.27) as comorbidity had an increased HR for readmission within 100 days after discharge (Table 2). When adjusting these comorbidities in the same model, diabetes mellitus and PAD lost their significance for the risk of readmission, i.e. only CKD, atrial fibrillation or COPD remained as factors associated with an increased risk of readmission within 100 days after discharge.

**Table 2:** Competing risk regression for readmissions within 100 days after discharge in HF patients with different comorbidities.

	HF	AF	PAD	CKD	DM	COPD	MM	
							1.09 (0.97–1.23)	**DM**
	1	1.22 (1.09–1.37)	1.28 (1.03–1.61)	1.29 (1.12–1.48)	1.13 (1.00–1.27)	1.17 (1.03–1.34)	1.22 (0.97–1.54)	**PAD**
							1.23 (1.10–1.38)	**AF**
							1.25 (1.09–1.44)	**CKD**
							1.17 (1.02–1.33)	**COPD**
*Age (years)*								
< 50	1	1	1	1	1	1	1	
50–75	1.38 (0.78–2.43)	1.32 (0.74–2.34)	1.37 (0.77–2.41)	1.40 (0.79–2.48)	1.36 (0.77–2.41)	1.33 (0.75–2.36)	1.27 (0.72–2.25)	
>75	1.47 (0.84–2.60)	1.38 (0.78–2.43)	1.46 (0.83–2.57)	1.52 (0.86–2.69)	1.48 (0.84–2.61)	1.44 (0.81–2.54)	1.36 (0.77–2.42)	
*Gender*								
women	1	1	1	1	1	1	1	
men	1.15 (1.03–1.28)	1.13 (1.01–1.26)	1.14 (1.03–1.27)	1.13 (1.01–1.26)	1.14 (1.02–1.27)	1.15 (1.03–1.28)	1.10 (0.99–1.23)	
*HF-subgroup*								
HFrEF	1	1	1	1	1	1	1	
HFmrEF	0.92 (0.78–1.10)	0.91 (0.77–1.09)	0.92 (0.78–1.10)	0.92 (0.77–1.09)	0.93 (0.78–1.10)	0.92 (0.77–1.09)	0.90 (0.76–1.07)	
HFpEF	1.16 (0.99–1.35)	1.13 (0.97–1.32)	1.16 (0.99–1.35)	1.14 (0.98–1.33)	1.15 (0.99–1.35)	1.14 (0.98–1.33)	1.11 (0.95–1.29)	
HF-NDP	0.97 (0.84–1.13)	0.96 (0.83–1.11)	0.98 (0.84–1.13)	0.98 (0.84–1.13)	0.98 (0.84–1.13)	0.96 (0.83–1.12)	0.95 (0.82–1.11)	
*NT-proBNP*								
HF unlikely	1	1	1	1	1	1	1	
"Grey zone"	1.16 (1.01–1.33)	1.11 (0.96–1.28)	1.15 (1.01–1.33)	1.16 (1.01–1.33)	1.16 (1.01–1.33)	1.17 (1.02–1.34)	1.12 (0.97–1.29)	
HF likely	1.52 (1.33–1.75)	1.47 (1.28–1.69)	1.52 (1.32–1.74)	1.51 (1.31–1.73)	1.53 (1.33–1.75)	1.53 (1.33–1.75)	1.47 (1.27–1.68)	
*Renal function (ml/min)*								
eGFR ≥ 60	1	1	1	1	1	1	1	
eGFR 30–59	1.15 (1.01–1.30)	1.14 (1.00–1.29)	1.14 (1.00–1.30)	1.08 (0.95–1.24)	1.14 (1.00–1.30)	1.15 (1.01–1.31)	1.08 (0.94–1.23)	
eGFR < 30	1.29 (1.09–1.53)	1.29 (1.09–1.52)	1.29 (1.09–1.52)	1.07 (0.88–1.31)	1.27 (1.07–1.50)	1.30 (1.10–1.54)	1.08 (0.89–1.32)	

Note; Hazard ratio with 95% confidence interval. HF = heart failure, HFrEF = heart failure with reduced ejection fraction, HFmrEF = heart failure with mildly reduced ejection fraction, HFpEF = heart failure with preserved ejection fraction, HF-NDP = heart failure with no defined subgroup, NT-proBNP = natriuretic terminal pro brain natriuretic peptide, AF = atrial fibrillation, CKD = chronic kidney disease, VHD = valvular heart disease, PAD = peripheral artery disease, IHD = ischemic heart disease, AMI = acute myocardial infarction, CVI = cerebrovascular insult, VHD = valvular heart disease, COPD = chronic obstructive pulmonary disease, DM = diabetes mellitus, HTN = hypertension, MM = multimorbidity, eGFR = estimated glomerular filtration rate (ml/min).

58% of the HF patients had atrial fibrillation as comorbidity, thus representing the most prevalent comorbidity causing an increased risk for readmissions in our study (Tables 1 and 2). Hypertension was the most prevalent (75%) comorbidity in HF patients in the current study, but these patients had no increased risk for readmissions. PAD, however, as the smallest patient group of comorbidity, constituting only 5% of HF patients, had an increased risk for readmissions within 100 days after discharge (Tables 1 and 2). Coronary artery disease is recognized as the main etiological factor in more than 50% of HF patients in North America and Europe [19], but the comorbidities ischemic heart disease or acute myocardial infarction had no impact on the risk for readmission within 100 days after discharge. Neither did the HF patients with CVI or valvular heart disease as comorbidity (Tables 1 and 2). For a more extensive description of this study population, please see the Table 1 in a prior study [20].

**Fig 1:**
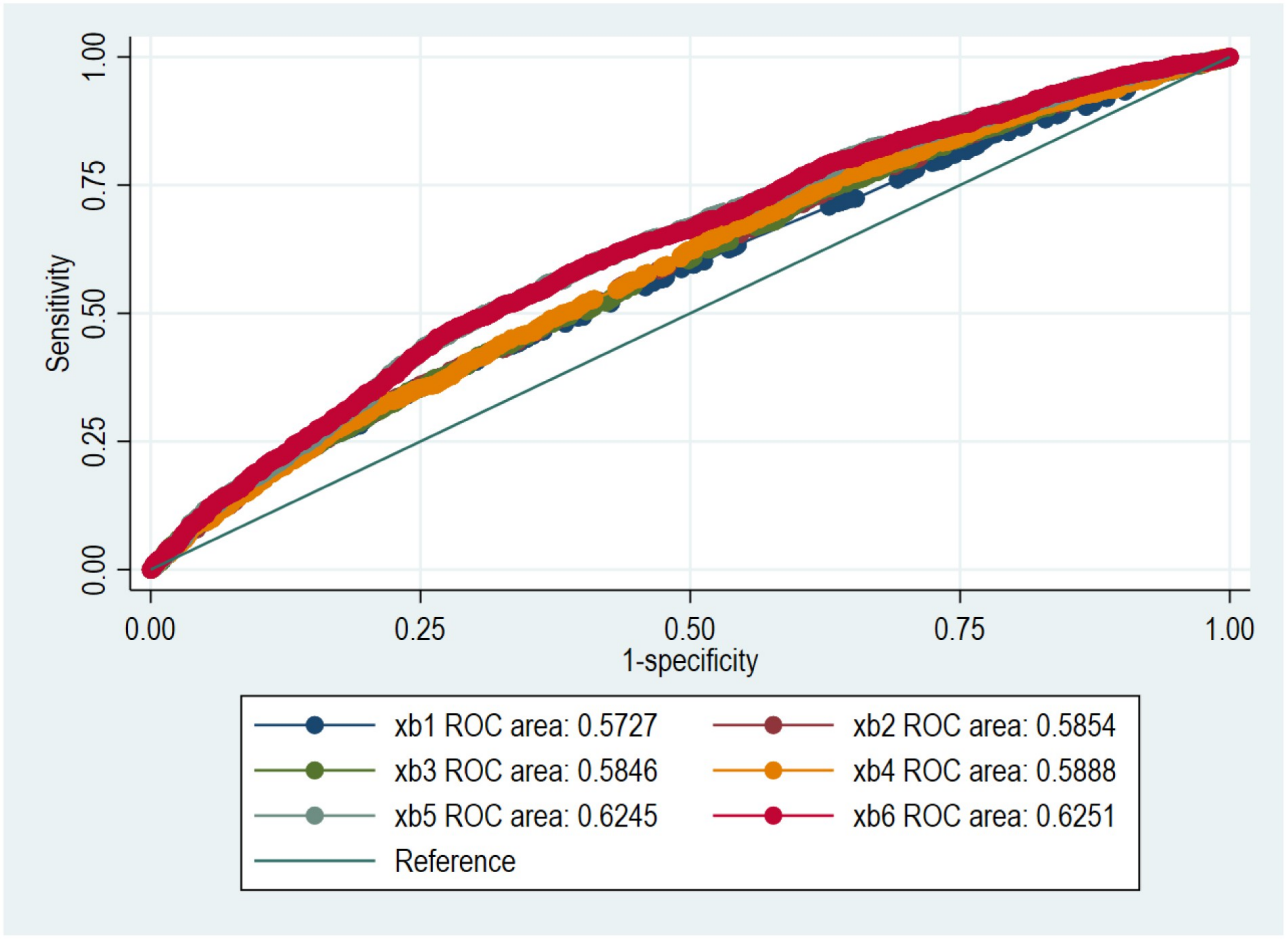
The receiver operating characteristic curve (ROC) and area under curve (AUC) of the probability of death or cardiovascular-related readmission within 100 days after discharge in the following models: xb1, xb2, xb3, xb4, xb5, xb6. Individual comorbidity level was calculated using logistic regression with the comorbidities as dichotomous indicator variables.

**Fig 2:**
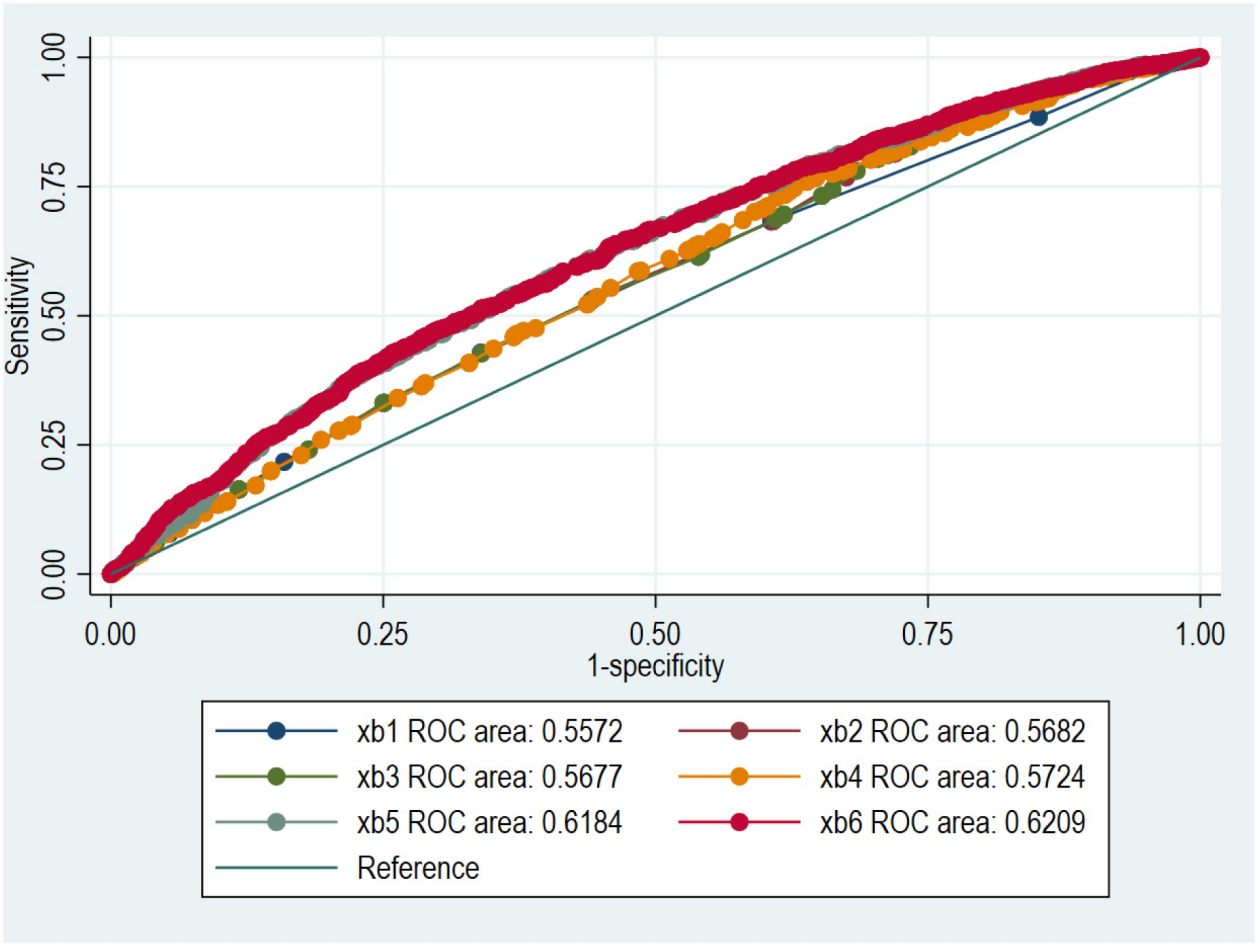
The receiver operating characteristic curve (ROC) and area under curve (AUC) of the probability of death or cardiovascular-related readmission within 100 days after discharge in the following models: xb1, xb2, xb3, xb4, xb5, xb6. Individual comorbidity level was calculated using Rasch analysis.

Logistic regression was performed with models of increasing complexity using the comorbidities as dichotomous indicator variables or by constructing a comorbidity measure of the 10 comorbidities using Rasch analysis. ROC analysis after the univariate logistic regression using the comorbidities as dichotomous indicator variables or Rasch analysis to estimate individual comorbidity level was 0.57 (95% CI 0.55–0.59) (xb1, Fig 1) and 0.56 (95% CI 0.54–0.57) respectively (xb1, Fig 2). AUC was significantly improved by adding the variables NT-proBNP and renal function (xb5, xb6, Figs 1 and 2). ROC analysis after the most complete models using logistic regression with the comorbidities as dichotomous indicator variables or Rasch analysis had an AUC of 0.63 (95% CI 0.61–0.64) and 0.62 (95% CI 0.60–0.64), respectively.

## Discussion

The present study included individuals admitted to hospital with a HF diagnosis and closely monitored these patients for 100 days post-discharge to determine whether presence of common comorbidities affected the risk of readmission. The comorbidities atrial fibrillation, CKD or COPD had an increased HR when adjusted in the same model, which explained the increased risk for readmission in HF patients with diabetes mellitus or PAD, regardless of age, gender, HF-subgroup and renal function. These results highlight the significance of the comorbidities atrial fibrillation, CKD or COPD in HF patients for the risk of cardiovascular related readmission. ROC analysis after logistic regression with comorbidities as dichotomous indicator variables and Rasch analysis to estimate individual comorbidity level was comparable, but the predictive value in the complete models was low.

Atrial fibrillation was reported to be an independent risk factor for HF readmission [21]. This is likely due to many shared pathophysiological mechanisms in both conditions and their propensity to exacerbate each other [22, 23]. Atrial fibrillation was found to be the most common comorbidity (58%) causing an increased risk for HF readmission in our study. In addition, patients with atrial fibrillation and concomitant HF have a poorer prognosis than patients with only one of these diseases [23]. Even HF patients with COPD were reported to have a higher mortality rate compared to patients with only one of these diagnoses [24, 25], which is in line with our results as only 67% of the COPD patients with HF were over 75 years old. HF treatment options could be limited among patients with CKD as a comorbidity and, thus, indirectly cause HF exacerbation and increased risk for cardiovascular-related readmission.

Ischemic heart disease is recognized as the main etiological factor in more than 50% of HF patients in North America and Europe [26], but the HF patients with acute myocardial infarction as comorbidity had a lower prevalence (61%) over 75 years than other comorbidities in our study. This patient category did not show an increased risk for readmission within 100 days after discharge, possibly due to their post-infarction follow up visits and high mortality rate as described by Solomonchuk et al. [27]. Nevertheless, the HF patients with CVI as comorbidity had the highest prevalence (80%) in the age group over 75 years, but no impact on the risk for readmission within 100 days after discharge, which suggests that this patient category is probably less related to cardiovascular events than other comorbidities in our study.

PAD had an increased risk for readmissions within 100 days after discharge although this patient group constituted only 5% of the HF patients. A retrospective cohort study was conducted from 2005 to 2016 and a total of 1481 elderly patients were hospitalized with acute decompensation of HF and discharged [28]. In total, 207 (14%) of these patients had a diagnosis of PAD and had an increased risk of at least one HF readmission, both within 30 days and one year after discharge from the index hospitalization [28]. The pathophysiology likely involved a strong association with ischemic heart disease, which could enhance the risk for HF-related readmission significantly. These results could explain our findings of HF patients with PAD, who had an increased risk for readmissions within 100 days after discharge.

A cohort study was conducted to assess adverse outcomes attributable to non-cardiac comorbidities and to compare their impact on hospitalizations in a chronic HF population between 2009 and 2013 [29]. Approximately 2300 elderly patients were recruited including 41% HFrEF and 59% HFpEF. Totally 14 non-cardiac comorbidities were considered including PAD, cerebrovascular event, dementia, COPD, rheumatologic disorders, peptic ulcer disease, diabetes mellitus, liver disease, malignancy, CKD, psychiatric disorders, anemia, obesity and hypertension. An increasing number of non-cardiac comorbidities were associated with an elevated risk for all-cause mortality, all-cause hospitalization, HF hospitalization, and non-cardiovascular hospitalization. These findings were similar for HFrEF and HFpEF, which is consistent with our findings [29].

## Strengths and limitations

We used competing risk regression in our calculations, which took into account mortality during the study period. Competing risk regression is a more accurate method than cox regression as these study participants were endangered and several of their comorbidities were associated with an increased mortality rate. People with COPD and HF, for instance, had a 7-folded mortality rate compared to COPD patients without HF [24]. Logistic regression and Rasch analysis had no statistically significant difference when estimating the individual comorbidity level, indicating that our results of these analysis were reliable. The Rasch analysis, however, offered a more pedagogical way to present the individual comorbidity level. This study was an observational study through three years, which made these findings more reliable. This specific study places its primary emphasis on comorbidities and the readmission risks related to cardiovascular issues. The data utilized in this study has been refined and offers a comprehensive coverage of these aspects.

Many of the HF patients presumably had overlaps of several comorbidities, which could affect the risk for cardiovascular-related readmission and mortality rate than HF patients with only one comorbidity. The readmission could also be conferred by decompensated HF as a consequence of deficient compliance in the patients, independent of their comorbidities. We did not consider other comorbidities associated with HF, for example ventricular tachycardia, which have the propensity to increase the risk for cardiovascular-related readmissions as well [30]. Valvular heart disease, CKD, CVI and hypertension could appear without clinical symptoms and thus remain frequently underdiagnosed or become discovered by chance. We did not consider the severity of these comorbidities, which could have different implications on the risk for readmission in HF patients. Neither did we record the success of specific treatment target goal. While such information could have provided valuable insights for this study, it was not feasible within the constraints of this data collection. In Sweden, multiple care programs are available to manage various chronic illnesses. For instance, patients with a prior myocardial infarction undergo outpatient follow-up visits after hospital discharge. The risk of hospital readmission can be influenced by the quality of follow-up care and the patients’ care plan. Factors like prompt follow-up in primary health care play a crucial role.

## Conclusion

The increased risk for cardiovascular-related readmission within 100 days after discharge in HF patients with diabetes mellitus or PAD had no significance after adjusting for atrial fibrillation, CKD or COPD in the same model. Using two measures of individual comorbidity level did not show any statistical difference, but the predictive value was found to be low in the current study. This means that other factors than these comorbidities we studied are of more importance for reducing the risk of cardiovascular related readmission within 100 days after discharge in HF patients. When managing individuals with HF, it is crucial to recognize that comorbidities exhibit limited predictive value. Instead, healthcare providers should prioritize attention to other influential factors to effectively prevent readmissions within the critical 100-day post-discharge period.

## Supporting information

S1 Appendix (DOCX)
