# Supplementary material for: Comorbidities in heart failure patients that predict cardiovascular readmissions within 100 days—An observational study
Source: PLoS One. 2024 Jan 2;19(1):e0296527. doi: 10.1371/journal.pone.0296527 (PMC10760770; doi:10.1371/journal.pone.0296527)
Supplement: S1 Appendix — (DOCX) [file pone.0296527.s001.docx]

| Appendix-Table 1. ICD 10 codes for the comorbidities included in our study. | |
| --- | --- |
|  |  |
| Disease | ICD-10 codes |
|  |  |
| Heart failure | I110, I420, I423 - I432, I438, I500 - I501, I509 |
| Hypertension | I10 - I15 |
| Ischemic heart disease | I20 - I25 |
| Cerebrovascular insult | I739 |
| Atrial fibrillation | I48 |
| Diabetes mellitus | E10 - E14 |
| Chronic obstructive pulmonary disease | J40 - J44 |
| Chronic kidney disease | N17 -N19 |
| Acute myocardial infarction | I21 - I22 |
| Peripheral artery disease | I739 |
| Valvular heart disease | I06, I34-I35, Q230, Q231, Q233 |

| Appendix Table 2. Recommended N-terminal pro brain natriuretic peptide (NT-proBNP) cut-offs for heart failure diagnosis. | | | | | |
| --- | --- | --- | --- | --- | --- |
| **Cut-off levels (ng/L)** | | | | |  |
|  | |  |  |  |  |
|  | | **Age < 50 years** | **Age 50 - 75 years** | **Age > 75 years** |  |
|  | |  |  |  |  |
| HF unlikely | | < 300 |  |  |  |
|  | |  |  |  |  |
| "Grey Zone" | | 300 - 450 | 300 - 900 | 300 - 1800 |  |
|  | |  |  |  |  |
| HF likely | | >450 | > 900 | > 1800 |  |
|  | |  |  |  |  |
| HF = heart failure; NT-proBNP = N-terminal pro brain natriuretic peptide | | | | | |
|  | | | | | |

| Appendix Table 3. Models xb1 - xb6 used for receiver operating characteristic (ROC) and area under curve (AUC). |
| --- |

| **Model** | **Variables** |
| --- | --- |
|  |  |
| xb1 | comorbidities |
| xb2 | comorbidities, age |
| xb3 | comorbidities, age, gender |
| xb4 | comorbidities, age, gender, HF-phenotype |
| xb5 | comorbidities, age, gender, HF-phenotype, NT-proBNP |
| xb6 | comorbidities, age, gender, HF-phenotype, NT-proBNP, renal function |

Appendix Figure. A flow chart of the study.


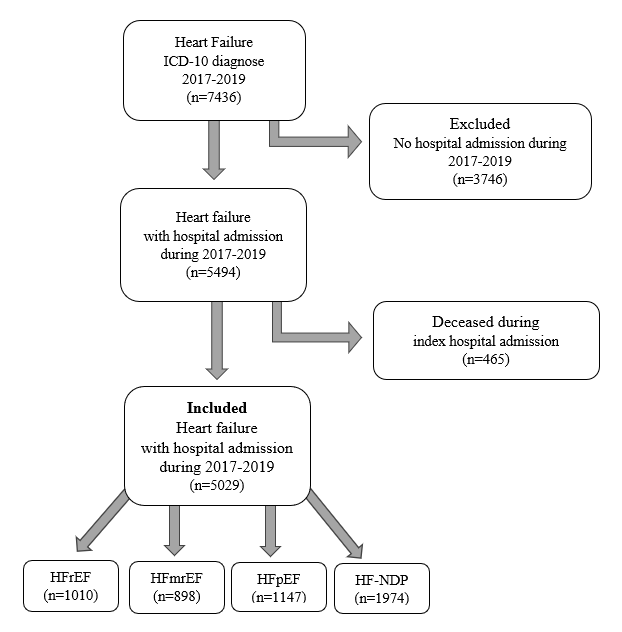


Note; HFrEF = heart failure with reduced ejection fraction, HFmrEF = heart failure with mildly reduced ejection fraction, HFpEF = heart failure with preserved ejection fraction, HF-NDP= heart failure with no defined phenotype, n= number of patients
